# Identification of grapevine clones via high-throughput amplicon sequencing: a proof-of-concept study

**DOI:** 10.1093/g3journal/jkad145

**Published:** 2023-07-03

**Authors:** Claudio Urra, Dayan Sanhueza, Catalina Pavez, Patricio Tapia, Gerardo Núñez-Lillo, Andrea Minio, Matthieu Miossec, Francisca Blanco-Herrera, Felipe Gainza, Alvaro Castro, Dario Cantu, Claudio Meneses

**Affiliations:** UC Davis-Chile, Life Sciences Innovation Center, Santiago 7520424, Chile; Centro de Biotecnología Vegetal, Facultad de Ciencias de la Vida, Universidad Andrés Bello, Santiago 8370186, Chile; Centro de Genómica y Bioinformática, Facultad de Ciencias, Ingeniería y Tecnología, Universidad Mayor, Santiago 8580745, Chile; Centro de Biotecnología Vegetal, Facultad de Ciencias de la Vida, Universidad Andrés Bello, Santiago 8370186, Chile; UC Davis-Chile, Life Sciences Innovation Center, Santiago 7520424, Chile; Centro de Biotecnología Vegetal, Facultad de Ciencias de la Vida, Universidad Andrés Bello, Santiago 8370186, Chile; Centro de Biotecnología Vegetal, Facultad de Ciencias de la Vida, Universidad Andrés Bello, Santiago 8370186, Chile; Departamento de Genética Molecular y Microbiología, Facultad de Ciencias Biológicas, Pontificia Universidad Católica de Chile, Santiago 8331150, Chile; Escuela de Agronomía, Facultad de Ciencias Agronómicas y de los Alimentos, Pontificia Universidad Católica de Valparaíso, Quillota 2263782, Chile; Department of Viticulture and Enology, University of California Davis, Davis, CA 95616-5270, USA; Center for Bioinformatics and Integrative Biology, Facultad de Ciencias de la Vida, Universidad Andrés Bello, Santiago 8370186, Chile; Wellcome Centre for Human Genetics, University of Oxford, Oxford OX3 7BN, UK; Centro de Biotecnología Vegetal, Facultad de Ciencias de la Vida, Universidad Andrés Bello, Santiago 8370186, Chile; ANID—Millennium Science Initiative Program—Millennium Nucleus for the Development of Super Adaptable Plants (MN-SAP), Santiago 8331150, Chile; Center for Research and Innovation, Viña Concha y Toro S.A, Pencahue, Talca 3460000, Chile; UC Davis-Chile, Life Sciences Innovation Center, Santiago 7520424, Chile; Department of Viticulture and Enology, University of California Davis, Davis, CA 95616-5270, USA; ANID—Millennium Science Initiative Program—Millennium Nucleus for the Development of Super Adaptable Plants (MN-SAP), Santiago 8331150, Chile; Departamento de Fruticultura y Enología, Facultad de Agronomía e Ingeniería Forestal, Pontificia Universidad Católica de Chile, Santiago 7820436, Chile; Departamento de Genética Molecular y Microbiología, Facultad de Ciencias Biológicas, Pontificia Universidad Católica de Chile, Santiago 8331150, Chile; ANID—Millennium Science Initiative Program Millenium Institute Center for Genome Regulation, CRG, Santiago 8331150, Chile

**Keywords:** *Vitis vinifera*, clonal genetic diversity, DNA fingerprinting, Plant Genetics and Genomics

## Abstract

Wine cultivars are available to growers in multiple clonal selections with agronomic and enological differences. Phenotypic differences between clones originated from somatic mutations that accrued over thousands of asexual propagation cycles. Genetic diversity between grape cultivars remains unexplored, and tools to discriminate unequivocally clones have been lacking. This study aimed to uncover genetic variations among a group of clonal selections of 4 important *Vitis vinifera* cultivars: Cabernet sauvignon, Sauvignon blanc, Chardonnay, and Merlot, and use this information to develop genetic markers to discriminate the clones of these cultivars. We sequenced with short-read sequencing technology the genomes of 18 clones, including biological replicates for a total of 46 genomes. Sequences were aligned to their respective cultivar's reference genome for variant calling. We used reference genomes of Cabernet sauvignon, Chardonnay, and Merlot and developed a de novo genome assembly of Sauvignon blanc using long-read sequencing. On average, 4 million variants were detected for each clone, with 74.2% being single nucleotide variants and 25.8% being small insertions or deletions (InDel). The frequency of these variants was consistent across all clones. From these variants, we validated 46 clonal markers using high-throughput amplicon sequencing for 77.7% of the evaluated clones, most of them small InDel. These results represent an advance in grapevine genotyping strategies and will benefit the viticulture industry for the characterization and identification of the plant material.

## Introduction

Grapevines are clonally propagated to preserve the cultivar's genetic, enological, and agronomic traits. However, mutations occur, giving rise to a vast diversity of clones or selections. Clones can exhibit differences in agronomic performance, including yield, berry weight, and the number of berries per cluster, as well as differences in the wine they produce, such as color, phenolic content, aromatic profile, wine acidity, and performance during bottle ageing (Wolpert *et al.* 1995; Farquhar and Clingleffer 2001; Benz *et al.* 2006; Burin *et al.* 2011; Dimovska *et al.* 2012; Šuklje *et al.* 2016).

Clonal differences in grapevines can be attributed to a cultivar of factors, including somatic mutations, epigenetic changes, and biotic determinants such as viruses (Franks *et al.* 2002). Somatic mutations, which occur during the growth and development of the plant, can be caused by several mechanisms. These include single base pair mutations, which are common in repetitive regions and can result from spontaneous deamination of methylated cytosine into thymine (Selker 1990; Mautino and Rosa 1998; Meunier *et al.* 2005; Vondras *et al.* 2019) as well as mutations in short sequence repeat (SSR or microsatellites) caused by polymerase slippage (Schlotterer and Tautz 1991). Somatic mutations can also lead to structural variations (SVs), like insertions, deletions, inversions, or translocations (Vondras *et al.* 2019; Zhou *et al.* 2019).

Traditionally, phenotypic characterization was the only tool to differentiate grapevine clones (Dounhovnikoff and Dodd 2003). However, phenotype-based strategies for clone discrimination in commercial settings are expensive and error prone (Imazio *et al.* 2002). Numerous initiatives have been undertaken to develop genetic markers capable of discriminating between clones to address the challenge of clone identification. These efforts have used features such as SSR, AFLP, and S-SAP. However, due to the limited genetic variability expected among clones, achieving complete resolution in clone identification has proven to be a persistent challenge. Additionally, these marker types have been utilized with limited acceptance, primarily due to concerns regarding their low reproducibility (Imazio *et al.* 2002; Riaz *et al.* 2002; Blaich *et al.* 2007; Stajner *et al.* 2009; Wegscheider *et al.* 2009; Pelsy *et al.* 2010). With next-generation sequencing (NGS) technologies, high-throughput analysis of single nucleotide polymorphisms (SNPs) or structural variants (SVs) is now possible, allowing for the simultaneous study of thousands of nucleotide positions (Garrido-Cardenas *et al.* 2018). NGS-based genotyping has proven invaluable for selecting and certifying plant material (Monte-Corvo *et al.* 2001), helping to prevent false certifications and denominations based solely on morphological analysis (Imazio *et al.* 2002 ). SNPs and SVs are a valuable source of genetic variability that can aid in accurately identifying and differentiating grapevine clones.

Over the past 15 years, several genomic initiatives have led to significant advancements in grape genomics. The release of the PN40024 and Pinot noir genomes in 2007 (Jaillon *et al.* 2007; Velasco *et al.* 2007) marked a significant milestone in the field; since then, new grapevine genome assemblies with higher contiguity and better representation of the heterozygosity have been published for a cultivar of cultivars, including Cabernet sauvignon (CS), Cabernet franc, Carmenere, Chardonnay (CH), and Zinfandel (Chin *et al.* 2016; Minio *et al.* 2019a and 2022; Roach *et al.* 2018; Vondras *et al.* 2019; Zhou *et al.* 2019). High-quality draft genomes allow scanning at the single-base resolution for genetic variability among clones within a cultivar. For example, 15 CH clones were sequenced, yielding 1,620 SNV (single nucleotide variant) markers (Roach *et al.* 2018). Similarly, an analysis of 16 Zinfandel clones found that most shared variants between clones were in nonrepetitive intergenic regions, while unique heterozygous sites were mainly associated with repetitive regions (Vondras *et al.* 2019). A study of Nebbiolo clones led to the discovery of 10 SNVs that could be used for clone identification (Gambino *et al.* 2017).

This study aimed to address the need for reliable methods to differentiate between clones by developing a new approach using high-throughput sequencing. Our main objective was to investigate whether amplicon sequencing could accurately identify different clones. Using short-read sequencing, we first analyzed the genetic variations among a selection of clones of economically significant grape cultivars [CS, Merlot (M), CH, and Sauvignon blanc (SB)]. We aligned the short-read sequences to existing genome references for CS, M, and CH. A new genome reference draft for SB was generated using long-read sequencing. The genetic variation information was then utilized to design primers for selectively amplifying polymorphic sites through short-read sequencing. The novelty of this work lies in the utilization of high-throughput amplicon sequencing, enabling the identification of clones with unprecedented efficiency. This approach allows for the simultaneous evaluation of up to 384 samples of each cultivar, limited only by the number of index combinations. By harnessing this technology, we have significantly reduced costs and improved the reproducibility of clone identification. Furthermore, we have released a new high-quality draft of the SB genome, further enhancing the scientific contributions of this study.

## Materials and methods

### Plant material

Forty-six plants from 18 clones of CS, M, CH, and SB were evaluated (Table 1). We collected young leaves to extract genomic DNA from different fields located between the regions of Valparaiso (*33°03′47″S 71°38′22″O*) and Maule (*35°25′36″S 71°40′18″O*) in the central Chilean valley. The evaluated clones were carefully selected based on their significance for the Chilean wine industry. Each biological replicate of a clone was randomly chosen from the commercial vineyards. Detailed information regarding the main characteristics of these clones can be found in Supplementary Material 1.

**Table 1.** Sequencing and mapping metrics for each clone of CH, SB, CS, and M.

Cultivar	Clone ID	Number of replicates	Raw data reads	Standard deviation	Post trimming reads	Mapped reads (%)
CH	4	3	181,770,528	15,423,112	136,372,000	99.2
CH	76	3	168,057,446	7,709,352	136,372,000	99.2
CH	95	3	169,459,674	15,852,831	136,372,000	99.2
CH	548	3	165,340,862	4,134,284	136,372,000	99.0
SB	1	2	165,529,797	3,712,968	117,651,540	98.8
SB	107	2	214,706,636	71,277,897	119,020,325	98.8
SB	159	3	172,666,060	6,129,927	117,468,275	98.9
SB	242	2	180,049,374	14,426,680	117,659,807	98.8
SB	530	3	177,520,481	3,987,649	118,485,608	98.9
CS	c46	2	142,245,783	21,641,720	122,438,400	97.4
CS	169	2	182,049,367	579,812	122,126,200	97.4
CS	170	2	152,923,714	13,861,080	122,665,906	97.2
CS	338	3	162,377,009	4,033,627	122,350,518	97.4
CS	412	3	156,783,251	13,325,975	122,955,261	97.5
M	181	3	136,266,544	2,710,124	103,685,230	99.8
M	346	1	149,900,404	-	103,745,200	99.5
M	347	3	140,755,284	4,983,903	103,685,230	99.7
M	348	3	130,844,937	26,672,549	103,685,230	99.7

Location of the plant material and metrics of sequencing data of the clone replicates. “Raw data reads” correspond to the average reads obtained by genome sequencing of each clone replicate. “Post-trimming reads” are the average reads that passed the trimming process. “Mapped reads” correspond to the percentage of reads mapped to CH, SB, CS, and M genome assembly.

### Sequencing, assembly, scaffolding, and annotation of a SB genome reference

High-quality genomic DNA was isolated from SB clone 1 (SB cl. 01) leaves using the method described by Chin *et al.* (2016). DNA purity was evaluated with a Nanodrop 2000 spectrophotometer (Thermo Scientific, Hanover Park, IL), DNA quantity with Qubit 2.0 Fluorometer (Invitrogen, Oregon, USA) with a Qubit dsDNA BR Assay Kit (Invitrogen), and integrity by electrophoresis. For single molecule, real-time (SMRT) sequencing, SMRTbell libraries were prepared as described in Chin *et al.* (2016); library quantity and quality were evaluated using a Bioanalyzer 2100 (Agilent Technologies, CA) and sequenced on a PacBio RS II (DNA Technology Core Facility, University of California, Davis).

De novo assembly of SB cl. 01 was performed at DNAnexus (Mountain View, CA, USA) using PacBio RS II data and the FALCON-unzip v1.7.7 pipeline (Chin *et al.* 2016). Repetitive content was masked in the reads before and after error correction using TANmask and REPmask modules in Damasker. Assembly was performed with FALCON-Unzip v1.7.7 (Chin *et al.* 2016), testing multiple parameters to produce the least fragmented assembly. These conditions are listed in Supplementary Material 2. Haplotype reconstruction was performed with default parameters. Finally, contigs were polished with Quiver (Pacific Biosciences, bundled with FALCON-unzip v1.7.7). Primary assembly underwent a scaffolding procedure to reduce sequence fragmentation. Primary contigs were scaffolds with SSPACE-LongRead v1.1 (Boetzer and Pirovano 2014), allowing junctions supported at least from 20 reads (-l 20). Hybrid scaffolding was then carried out with Hi-C (Dovetail Genomics, Scotts Valley, CA, USA) using the proprietary HiRise software v1.3.0-1233267a1cde.

Repeat and gene annotation were performed, as reported by Vondras *et al.* (2019). RepeatMasker v4.0.6 (Smit *et al.* 2013) was loaded with a custom *Vitis vinifera* repeat library (Minio *et al.* 2019b ), which was used to identify repetitive elements in the genome. To annotate the genes, publicly available datasets were used as evidence for gene prediction. Transcriptional evidence included *Vitis* ESTs, CS corrected Iso-Seq reads, Tannat, Corvina, and CS transcriptomes and previously published RNA-seq data for SB (PRJNA260535). The SwissProt viridiplantae data and *Vitis* data were used as experimental evidence. Each RNA-seq sample was trimmed with Trimmomatic v0.36 (Bolger *et al.* 2014) and assembled with Stringtie v1.3.3 (Pertea *et al.* 2015). These data were then aligned to the genome draft using Exonerate v2.2.0 (transcripts and proteins) (Slater and Birney 2005) and PASA v2.1.0 (transcripts) (Haas *et al.* 2003). Alignments and ab initio predictions generated with SNAP v2006-07-28 (Korf 2004), Augustus v3.0.3 (Stanke *et al.* 2006), and GeneMark-ES v4.32 (Lomsadze *et al.* 2005) were used as input for EVidenceModeler v1.1.1 (Haas *et al.* 2008). EVidenceModeler was used to identify consensus gene structures. Functional annotation was obtained by integrating homology with the RefSeq plant protein database (https://ftp.ncbi.nlm.nih.gov/refseq/, retrieved 2017 January 17) and as described in Jones *et al.* (2014).

### Illumina library construction and sequencing

Genomic DNA was extracted from 1 g of leaf powder in liquid nitrogen using the commercial kit DNeasy Plant Mini Kit QIAGEN (QIAGEN, Düsseldorf, Germany) following the manufacturer's indications. Quantification was done using a Qubit 2.0 Fluorometer (Invitrogen, Oregon, USA) with a Qubit dsDNA BR Assay Kit (Invitrogen). The genomic DNA integrity was evaluated by 0.8% agarose gel.

The construction of the 46 libraries was performed using the TruSeq Nano DNA Kit (Illumina, CA, USA) following the manufacturer's protocol. The library integrity was evaluated by capillary electrophoresis using the Fragment Analyzer Automated CE System (Analytical Advanced Technologies, Iowa, USA) with the DNF-474 High Sensitivity NGS Fragment Analysis Kit (Analytical Advanced Technologies), according to the manufacturer's instructions. Finally, 46 libraries were sequenced in paired-end of 150-bp length reads on the Illumina HiSeq2500 by the Macrogen Sequencing Service (Seoul, South Korea).

### Variant calling

The raw sequences were analyzed using FastQC v0.11.7 (Andrews 2010), followed by a coverage standardization of 20×. To do this, 137,372,000 reads were kept from each clone genome in CH, 119,020,000 in SB, 124,600,000 from CS, and 103,685,230 in M clones using the software seqtk v1.3-r106 (https://github.com/lh3/seqtk). Trimming was performed using Trim-galore software v0.5.0 with PHRED quality threshold *Q* > 25 (Krueger 2012). Each clone genome was mapped to the genome assembly of its cultivar using the primary assembly. The genome mapping was performed with bwa-mem software v0.7.17-r1188 (Li *et al.* 2008). Before the variant calling process, the mapped genome sequence reads were sorted using Samtools software v1.9 (Li *et al.* 2009) and prepared with Picard-tools software v2.16.1 using the AddOrReplaceReadGroups, MarkDuplicates, and CleanSam commands (https://broadinstitute.github.io/picard/).

We used GATK HaplotypeCaller v4.0.9.0 (Mckenna *et al.* 2010) to perform the variant calling of each clone genome using the primary assembly of SB and CH clones (Zhou *et al.* 2019). In CS, the primary assembly version was the one described by Chin *et al.* 2016, while in M clones, it was the primary assembly described by Massonnet *et al.* 2020. Two different variant calling protocols were used: first on each sample individually and second with a joint genotyping step combining all samples following the GATK best practices (available at https://gatk.broadinstitute.org). A variant quality filter of *Q* > 100 was applied for both protocols. The global distribution of variants detected in all clones was evaluated by a Circos plot (Krzywinski *et al.* 2009). Variants and gene densities were calculated in 100-kbp windows for plotting. Only variants consistently present in each clone's replicates were used for principal component analysis (PCA). To identify clone-specific variants, we extracted variants that were present in all replicates of a clone and absent in all the other samples.

PCA plots were generated in R v3.5.3 with the R packages factoextra v1-0-5 and FactoMineR v1.4.1. Predicted functional effects were estimated using the software SnpEff v4.3t (Cingolani *et al.* 2012).

### Unique variant validation by custom high-throughput amplicon sequencing

High-quality, unique variants were selected based on variant quality assigned by GATK. Selected variants were also evaluated by visualizing the read mappings with the Integrative Genome viewer software IGV v2.5.3. Primers were designed to amplify the 141–487-bp region (average equal to 201.8 bp), flanking the variant site using Primer3. To perform the marker validation, a 2-step PCR protocol was designed. In the first amplification, specific primers were used for the region of interest, including the candidate marker. These primers also contain a tail that hybridizes with primers used in the second PCR step. In the second amplification, primers were used containing adapters to perform the amplicon sequencing. The primers used in this protocol are listed in Supplementary Material 3. The first PCR was performed in a total volume of 15 *µ*L, including 3 *µ*L of each specific primer (1 *µ*M), 1.5 *µ*L of DNA (5 ng/*µ*L), and 7.5 *µ*L of Taq Polymerase SapphireAmp Fast PCR master mix (Takara). The first PCR cycle was an incubation at 94°C for 1 min followed by 25 cycles of 5 s at 98°C, 5 s at the specific annealing temperature of each primer, 5 s at 72°C, and a final 5 min incubation at 72°C. A purification step by magnetic beads followed (AMPure XP, Beckman Coulter), adding 10 *µ*L of free nuclease water and 20 *µ*L of magnetic beads to each tube following the manufacturer's protocol. The second PCR was performed by adding 2 *µ*L of purified PCR product, 2 *µ*L of Illumina index (N7XX + S5XX, 10 *µ*M), 4 *µ*L of free nuclease water, and 10 *µ*L of SapphireAmp Fast PCR master mix. The amplification process consisted of a first incubation at 94°C per 1 min, followed by 8 cycles of 5 s at 98°C, 5 s at 68°C, 7 s at 72°C, and a final incubation at 72°C per 5 min. Libraries were purified by using magnetic beads. The size and integrity of the libraries were evaluated by capillary electrophoresis using the Fragment Analyzer Automated CE System (Analytical Advanced Technologies) with the DNF-474 High Sensitivity NGS Fragment Analysis Kit (Analytical Advanced Technologies), according to the manufacturer's instructions. Libraries were sequenced in paired-end of 150-bp length reads on the Illumina MiSeq.

## Results

### Whole genome resequencing of clones of CS, CH, M, and SB

We resequenced the genomes of 46 *V. vinifera* clones from the cultivars SB, CH, CS, and M, which were selected based on their importance to the wine industry worldwide. The clones were selected for each cultivar based on availability in the Chilean nursery germplasm. The genome of each biological replicate was sequenced separately and mapped individually against the genome assembly of their respective cultivar. We used biological replicates for each clone to differentiate intra- and interclonal genetic variations. We obtained between 130,844,937 and 214,706,636 raw reads for each clone, corresponding to an estimated average coverage of 20× (Table 1). Reads that passed the quality filter and trimming were aligned to the corresponding primary sequence of the reference genome available for CS (Chin *et al.* 2016), CH (Zhou *et al.* 2019), and M (Massonnet *et al.* 2020). In the case of SB, we developed a new reference genome. SB clone 01 (SB cl. 01) was sequenced at 122× coverage using single-molecule real-time (SMRT; Pacific Biosciences) technology. The long reads were assembled into primary contigs and haplotigs. The SB assembly has 358 scaffolds spanning 635.31 Mbp with an N50 scaffold length equal to 24.53 Mbp (Table 2). CS, CH, SB, and M registered a percentage of mapped reads above 97% (Table 1).

**Table 2.** Reference genome assembly and completeness metrics for CH, SB, CS, and M.

Genome characteristics	CH	SB	CS	M
Clone ID	FPS_04	SB cl. 01	08_ENTAV	181_ENTAV
Assembly length (Mbp)	605.96	635.31	591.42	606.51
Number of scaffolds	684	358	718	1,485
Maximum scaffold length (Mbp)	35.15	23.48	14.08	6.32
Scaffolds > 100 Kbp	355	255	525	1,046
Scaffolds > 1 Mbp	34	99	177	152
Scaffolds > 5 Mbp	22	46	15	3
N50 length (Mbp)	24.53	8.02	2.17	0.81
N50 scaffolds	11	26	72	204
Number of Ns (Mbp)	4.06	3.52	0	0.84
GC %	34.10	34.30	34.80	34.50
Complete BUSCOs (%)	96.80	99.30	95.00	98.30
Fragmented BUSCOs (%)	0.90	0.50	1.20	1.20
Missing BUSCOs (%)	2.30	0.20	3.80	0.50

Assembly metrics and BUSCO analysis result for each cultivar. “Complete BUSCOs” corresponds to the percentage of BUSCO genes found as complete with BUSCO v.5.2.2 software with viridiplantae_odb10 dataset in the CH (Zhou *et al*. 2019), SB (Zhou *et al*. 2019), CS (Chin *et al*. 2016), and M (Massonnet *et al*. 2020) primary reference genomes. Under the same logical order, “Fragmented BUSCOs” corresponds to partially found genes, and “Missing BUSCOs” to genes not found on the assembly.

### Genetic variability within and between clones

**Fig. 1.**
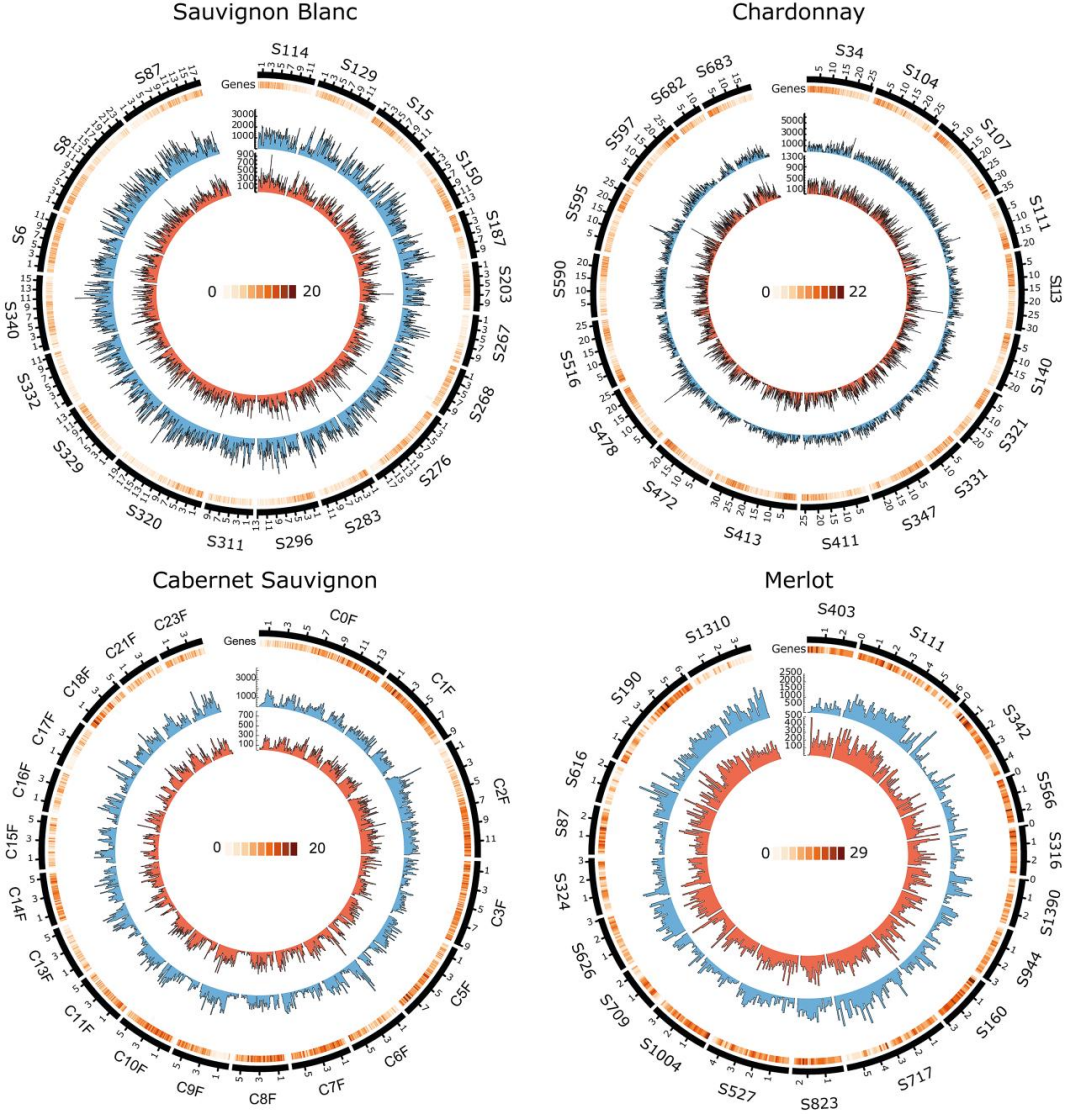
Frequency of genetic variant (SNV and InDel) genes and repetitive elements on the 19 largest contigs of SB, CH, CS, and M clones compared to their respective genome assemblies. The Circos diagram presents a hierarchical visualization, depicting genome scaffolds on the outer layer, gene frequency as a heatmap, and single nucleotide polymorphisms (SNPs) and insertions/deletions (InDels) as inner histograms.

Variant calling was performed for each biological replicate. At the SNV level, all cultivars exhibited comparable levels of variants relative to their respective reference genomes. The rate of SNV detected varied between 2.8 million in SB and 3.3 million in M. As expected, most detected variants were heterozygous (Table 3). The VCF files were deposited in a repository (https://doi.org/10.5281/zenodo.7938765). In InDels, the detected variants varied from 595,000 to 1.35 million, representing between 2.4 and 2.8 Mb of the respective reference genome. Their heterozygous percentage was lower and more variable than SNV, reaching values as low as 42.9% for M (Table 3). The low degree of heterozygosity detected in M may be due to false primary sequences (i.e. redundant homologous regions) in the reference (Minio *et al.* 2019a). SNV and InDel were mainly evenly distributed throughout the 19 largest contigs of each cultivar (Fig. 1). The highest variant density was found in regions with low gene density.

**Table 3.** DNA variant detection of each clone genome compared to the reference genome.

Cultivar	SNV				InDel			
	Number of average SNV	Het %	SNV/1kbp	Ts/Tv	Number of average InDel	Average InDel (Mb)	Het %	InDel/1 kbp
CH	2,855,928 ± 12,066	98.9	4.7	2.1	1,189,657 ± 12,971	2.81 ± 0.04	92.2	1.9
SB	2,804,443 ± 16,850	99.2	4.4	2.1	1,088,903 ± 18,200	2.66 ± 0.05	68.2	1.7
CS	2,988,447 ± 20,736	99.5	5.3	2.1	595,565 ± 7,277	2.49 ± 0.04	80.8	1.1
M	3,261,479 ± 83,429	97.9	5.5	2.1	1,340,800 ± 89,793	2.83 ± 0.29	42.9	2.2

Average SNV represents the total SNV detected by average among all the cultivar clones. The SNV frequency is represented by the number of SNV per 1 kbp. Ts/Tv corresponds to the transition/transversion rate. Average InDel compares to the total InDel average among the clone replicates detected in the different clone genomes. InDel frequency was represented by the number of InDel per 1 kbp. (Het, heterozygous).

**Fig. 2.**
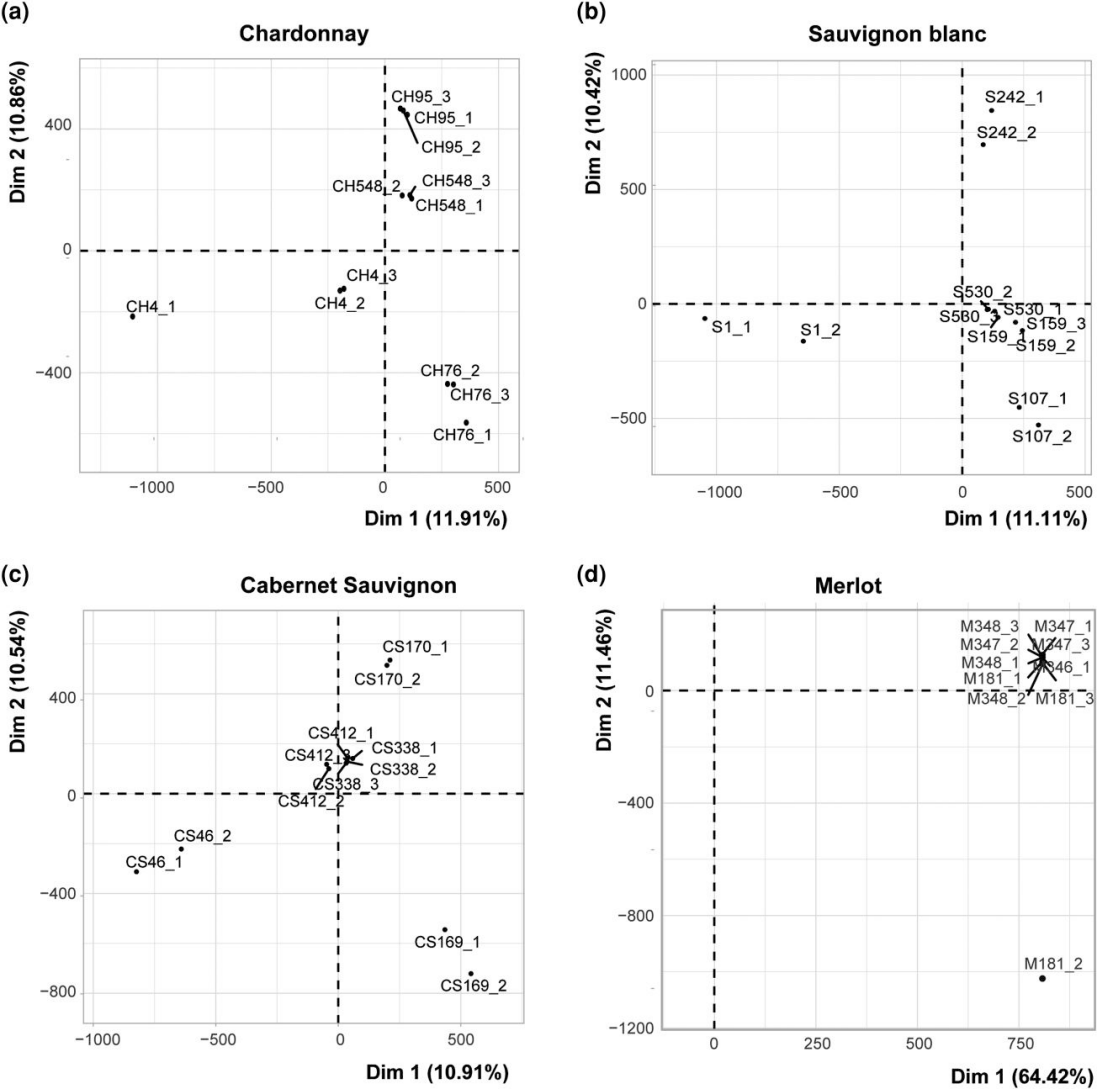
Genetic variability among *V. vinifera cv*. CH, SB, CS, and M clones. PCA of grapevine clones represents their genetic variability in dimensions 1 versus 2. Graphs A, B, C, and D represent the genetic variability among clones of CH, SB, CS, and M, respectively.

The genetic relation among clones was evaluated through joint variant genotyping, followed by PCA. We observed a clear separation among the 4 CH clones, with clustering of biological replicates (Fig. 2a). For SB, 3 out of 5 clones were separated from the others. However, clones 159 and 530 were the exception, located next to each other (Fig. 2b). CS presented a similar pattern, with clones c46, 170, and 169 being well separated and clones 338 and 412 that perfectly colocalized (Fig. 2c). The PCA for M was unsuccessful in separating the different clones, with all of them clustering together, suggesting little genetic variability between these particular M clones. The only exception was one of the replicates of clone 181, which is separated from all the clones by PC2 (Fig. 2d); an outlier replicate of clone 181 was discarded from further analysis. All those cases where PCA could not separate the clones suggest low genetic variability, making searching for SNPs or InDels that discriminate clones more challenging.

### Identification of clone-specific genetic variants

The next step was identifying unique variants shared between biological replicates but not between clones (Table 4). The number of unique variants differed significantly for SNV and InDel, ranging from 225 SNV in CH to 1,442 in SB, a 6-fold difference. The InDel analysis showed a similar trend, with CH having the lowest count of 50 InDels and CS the highest with 422, an 8-fold difference. This allowed grouping of the cultivars into 3 categories: CH with low values for both SNV and InDel, M with intermediate values, and CS and SB with high values. 94% of unique SNV variants in all clones were heterozygous. Most clones exhibit unique InDel variants that are over 85% heterozygous. However, the M clones display a different pattern, with a lower percentage of heterozygous InDel variants (68%) than the other cultivars (Table 4).

**Table 4.** Clone-specific variant identification.

Cultivar	Clone ID	SNV	Heterozygous %	Average heterozygous SNV	InDel	Heterozygous %	Average heterozygous InDel
CH	4	107	99.1	97.3 ± 1.72	26	84.6	87.35 ± 5.71
	76	270	98.1		78	87.1	
	95	289	95.1		44	95.4	
	548	233	96.9		51	82.3	
SB	1	2,009	98.4	99.42 ± 0.61	668	65.4	85.88 ± 12.14
	107	1,319	99.6		231	94.3	
	159	1,027	100.0		199	85.9	
	242	1,900	99.4		452	88.2	
	530	952	99.7		185	95.6	
CS	c46	2,077	98.1	97.06 ± 1.52	754	92.6	92.62 ± 2.01
	169	1,716	97.2		498	93.3	
	170	2,065	97.8		642	90.1	
	338	735	94.4		180	91.6	
	412	1,053	97.8		317	95.5	
M	181	306	99.0	98.25 ± 1.31	41	75.6	68.1 ± 34.8
	346	1,887	96.3		745	70.3	
	347	434	98.6		113	58.4	
	348	434	99.1		70	67.3	

The SNV and InDel correspond only to variants detected in all the replicates of 1 clone but absent in all the other clones.

### Validation of diagnostic loci using high-throughput amplicon sequencing

Considering the variant quality and using IGV software for visual verification, we selected specific sites for confirmation through custom high-throughput amplicon sequencing. Our analysis validated 48 genetic markers (11 SNV and 35 InDel) in 14 of the clones (74%), with an average of 3 markers per clone (Table 5).

**Table 5.** List of validated markers for the different cultivars.

Cultivar	Clone ID	Marker ID	DNA variant	Scaffold/start position (bp)	Allele length (bp)
CH	95	CH95_2	InDel	GC_411/17,831,79	180/190
		CH95_3	InDel	GC_595/23,803,897	194/201
		CH95_4	InDel	GC_677/30,351	229/240
CH	76	CH76_1	InDel	GC_478/6,101,20	461/487
		CH76_2	InDel	GC_413/11189702	325/344
		CH76_4	InDel	GC_472/14,106,797	238/243
CH	548	CH548_1	InDel	GC_413/33,977,545	392/399
		CH548_2	InDel	GC_595/10,809,953	225/235
		CH548_3	InDel	GC_140/4,753,007	247/256
		CH548_4	InDel	GC_648/23,853	146/154
SB	242	SB242_2	InDel	scaffold_340/10,773,980	181/156
		SB242_3	SNV G/A	scaffold_356/1,788,235	249
		SB242_5	SNV C/T	scaffold_326/3,712,137	209
SB	1	SB1_5	InDel	scaffold_347/8,417,924	175/164
		SB1_7	InDel	scaffold_312/849,432	183/180
		SB1_8	InDel	scaffold_20/302,238	195/186
		SB1_9	InDel	scaffold_348/441,728	250/220
SB	159	SB15_1	SNV G/A	scaffold_320/11,246,529	218
		SB159_3	InDel	scaffold_283/7,733,780	176/187
		SB159_5	SNV G/A	scaffold_187/9,263,435	167
SB	107	SB107_1	InDel	scaffold_87/7,203,360	221/217
		SB107_3	InDel	scaffold_340/7,767,043	240/242
		SB107_6	InDel	scaffold_11/1,681,496	245/230
SB	530	SB530_3	InDel	scaffold_271/195,088	182/176
		SB530_7	InDel	scaffold_8/17,091,159	246/255
		SB530_9	InDel	scaffold_283/9,918,472	188/175
CS	46	CS46_1	InDel	000028F/1,708,937	141/153
		CS46_2	InDel	000078F/2,184,324	156/168
		CS46_4	InDel	000028F/3,575,774	145/153
CS	169	CS169_2	SNV T/A	000119F/1,481,484	230
		CS169_3	SNV A/G	000280F/294,173	150
		CS169_4	InDel	000204F/233,732	194/195
CS	338	CS338_2	SNV C/T	000017F/732,204	165
		CS338_6	InDel	000212F/205,063	293/299
M	348	M348_1	InDel	GcS596/405,864	165/178
		M348_2	InDel	GcS648/645,734	235/238
		M348_3	SNV A/T	GcS472/1,346,008	180
		M348_4	SNV G/A	GcS111/3,645,198	250
M	181	M181_1	InDel	GcS645/107,657	244/249
		M181_2	InDel	GcS591/510,148	198/203
		M181_3	SNV A/G	GcS828/184,353	246
		M181_4	SNV C/T	GcS1310/197,649	212
M	346	M346_2	InDel	GcS1195/341,995	229/236
		M346_3	InDel	GcS1246/39,718	176/184
		M346_4	InDel	GcS1004/2,348,379	313/328
		M346_5	InDel	GcS145/215,538	176/192

List of markers validated for CH, SB, CS, and M clones. The validated markers correspond to heterozygous SNV and InDel.

**Fig. 3.**
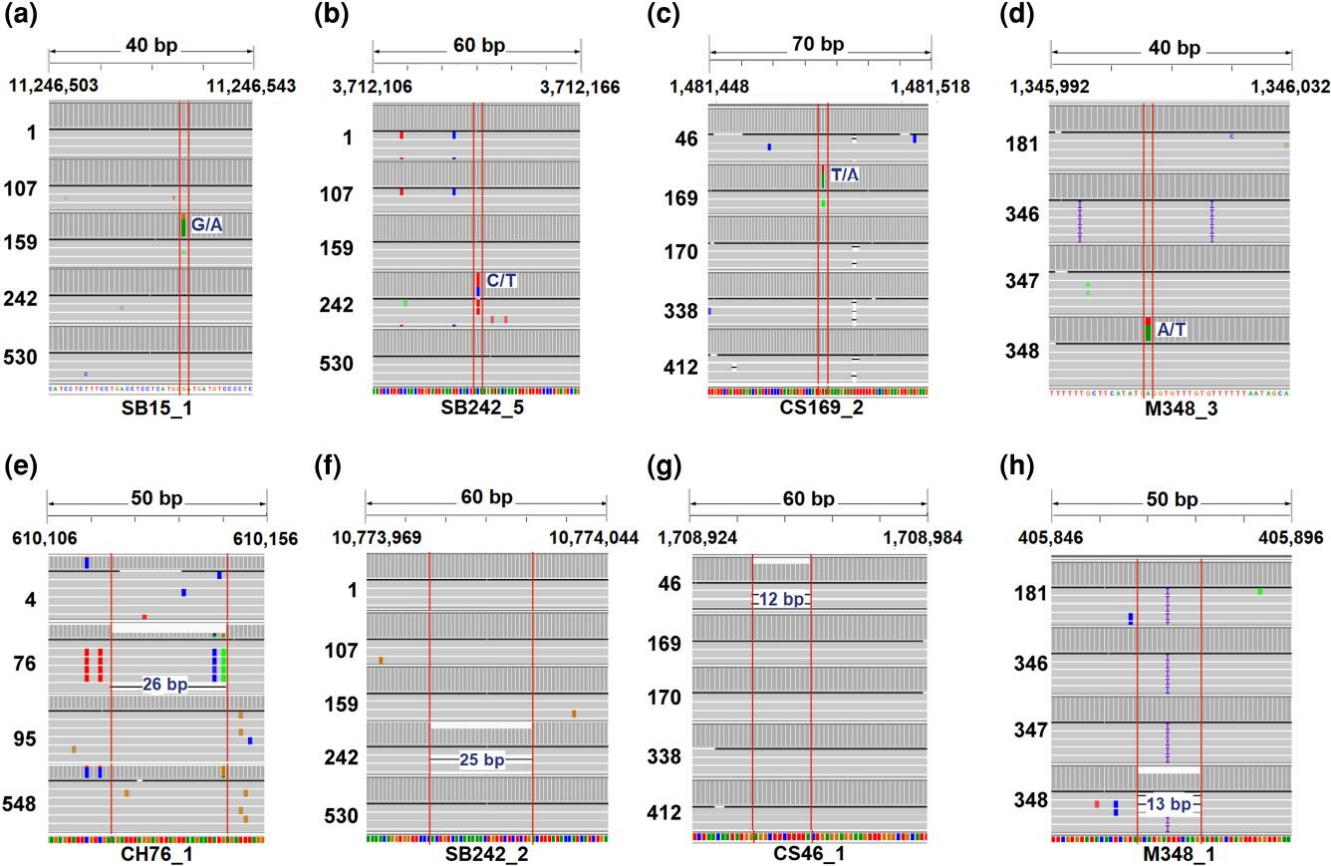
SNV and InDel markers validated by amplicon sequencing discriminate between 1 clone and the rest of the clones within 1 cultivar. Each panel shows the sequence alignment of DNA regions with selected clonal markers. a) Heterozygous SNV (G/A) was validated as a marker to discriminate between clone 159 and the rest of the clones of SB. b) Heterozygous SNV (C/T) used for SB clone 242. c) Heterozygous SNV (T/A) for CS clone 169. d) Heterozygous SNV (A/T) for M clone 348. e) Heterozygous InDel of 26 bp was used as a marker to discriminate between clone 76 and the rest of the clones of CH. f) Heterozygous InDel of 26 bp for SB clone 242. g) Heterozygous InDel of 12 bp for CS clone c46. h) Heterozygous InDel of 13 bp for M clone 348. The bar graphs represent the read coverage of each point.

We validated markers in 3 of 4 CH clones, 5 SB clones, 3 of 5 CS clones, and 3 of 4 M clones (Supplementary Material 3). Each marker distinguished 1 clone from the rest of the cultivar's clones. The validated markers were unique to the clone, initially detected by bioinformatic analysis, making them clone-specific variants. These markers can aid in selecting and tracking propagated plant material. They also allow direct identification of each clone as they are absent in other clones, and the presence of 1 marker suffices (Fig. 3). Most validated markers were InDels located in intergenic regions, averaging 10.5 ± 6.5 bp in length.

## Discussion

The grapevine has a wide cultivar of cultivars due to its long history of cultivation (This *et al.* 2006). These cultivars are propagated vegetatively to preserve their agronomic and enological traits, resulting in clonal selections. During propagation, some plants may exhibit phenotypic differences of commercial interest. These grapevine materials can be selected, multiplied, and cataloged as new clones after cultivation, agronomic, enological evaluation, and characterization. Several studies have explored clonal diversity in various cultivars such as Pinot Noir (Franks *et al.* 2002; Blaich *et al.* 2007; Wegscheider *et al.* 2009; Carrier *et al.* 2012; Vezzulli *et al.* 2012; Ocaña *et al.* 2013; Pelsy *et al.* 2015), CH (Bertsch *et al.* 2005; Roach *et al.* 2018; Zhou *et al.* 2019) and Zinfandel (Vondras *et al.* 2019). This study differs in methodology from previous ones, as it utilized amplicon sequencing, enabling high-throughput analysis of multiple samples. Another difference is that by using our strategy, we could detect 2 types of markers, SNVs and InDels. At the same time, previous analyses focused on only 1 such as AFLP, SVs, SNPs, InDels, mobile elements, microsatellite-sensitive amplified polymorphism, S-SAP, real-time single molecule sequencing, or resequencing data.

This is demonstrated the feasibility of using high-throughput amplicon sequencing to identify the genetic variation among multiple grapevine cultivars, which are critical to the viticulture industry. Our approach resulted in the development and validation of 46 genetic markers that allow for the discrimination of 14 clones from 4 important cultivars. This proof of concept highlights the potential of using an amplicon sequencing strategy to identify and track grapevine cultivars in the industry. We have developed a strategy to conduct high-throughput genotyping of up to 384 samples of each cultivar simultaneously within a single sequencing run (the available index combinations limit the number of samples). This represents a significant advancement in plant genotyping. The automated analysis process employed in our methodology reduces errors and enhances reproducibility compared to previous labor-intensive techniques such as SSR, AFLP, and S-SAP.

**Fig. 4.**
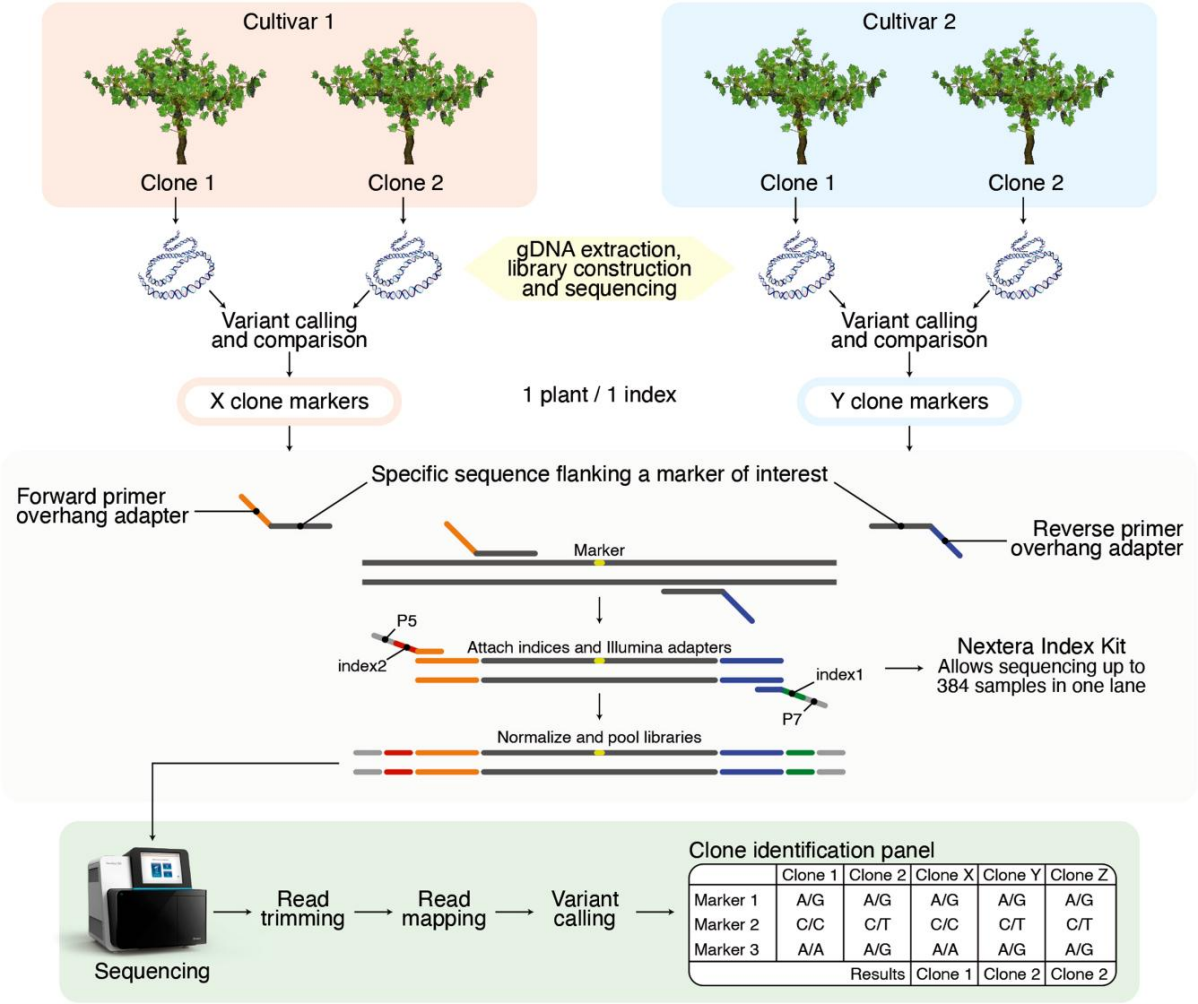
Clone identification strategy through high-throughput amplicon sequencing. All evaluated plants of each cultivar are genotyped using all the markers developed for the cultivar. A 2-step PCR assigns a unique index pair to each plant, and all the markers evaluated in 1 plant have the same index pairs. All the libraries are pooled and sequenced together, and then, each library is mapped to its respective reference genome. Then, the variants are called and a final filter is made to obtain only the markers, with which the clone identification panel is made.

Additionally, the number of samples that can be evaluated using our approach far surpasses those achievable with these traditional methods. Moreover, the ability to genotype many samples per sequencing run significantly reduces the costs per sample. Detailed information regarding the analysis process can be found in Fig. 4, which illustrates the step-by-step analysis, beginning with individual plants for each clone and culminating in the identification panel utilizing the combination of genotypes derived from the developed markers for each cultivar.

### Grapevine genotyping by whole genome sequencing

The grapevine genome is highly heterozygous and repetitive. The primary assembly of CS has a length of 591 Mbp, with the heterozygous regions of the genome represented by haplotigs covering 368 Mbp (Chin *et al.* 2016). Our results agreed with Chin *et al.* (2016), as 99.4% of SNVs and 80.8% of InDels were heterozygous when clone genomes were compared to their respective genome assemblies. These results were not unexpected given the high heterozygosity of grapevine and the fact that we used the haploid genome to perform the read mapping and variant calling.

The difference between SNV and InDel percentage could be associated with variant calling error, given that SNV calling is more accurate than InDel calling in terms of sensitivity, reported at around 90.2% for GATK HaplotypeCaller compared to SAMtools, Dindel, and Freebayes (Kim *et al.* 2017). The global frequency of SNVs detected in this study was lower than in previous studies, with 4.7 SNV/kbp in CH, 4.4 SNV/kbp in SB, 5.3 SNV/kbp in CS, and 5.5 SNV/kbp in M. This is in contrast to previous studies that reported higher levels of genetic variability, such as the comparative genomic analysis of wine and table grapes against the grapevine genome PN40024, which reported 3,732,107 SNVs (7.7 SNV/kbp) (Zhou *et al.* 2017), or the evaluation of 472 different grapevine accessions, which reported 12,549,273 total SNVs (Liang *et al.* 2019). However, it is necessary to consider that we are evaluating intracultivar genetic variability in our study and the other studies evaluated different accessions.

### Variants identified by whole genome resequencing allow differentiating clones

The genetic variability among grapevine clones was analyzed by comparing variants obtained from high-throughput amplicon sequencing. The variants were filtered to include only those shared among all replicates of each clone. PCA was used to visualize the genetic variability between the clones. Results showed that the variability between clones varied across cultivars. Clones of CH and SB showed clear separation in the PCA plot, while in CS, some clones were closely grouped, indicating that the differences between these clones may be at the epigenetic. In the case of M, a low genetic variability among clones was observed, being located all together except for biological replicate 2 of clone 181, which was completely separate from all the other samples. This sample was therefore excluded from further analyzes as it is suspected of being an error in the vineyard records. When clone-specific variants were compared, it was possible to identify a similar number of variants in those clones with low genetic variability compared to the clones separated in the PCA plot. The low genetic variability detected in the present study among CS and M clones suggests that other mechanisms could play a role in their phenotypic variation. These mechanisms involve transposable elements (Carrier *et al.* 2012), epigenetic variation (Ocaña *et al.* 2013), disease load (Franks *et al.* 2002), or more significant SVs (Zhou *et al.* 2019).

### Genetic markers for clone identification

Identifying genetic clones in the viticulture industry continues to be a challenge. Previous studies have used SSR markers (Riaz *et al.* 2002; Pelsy *et al.* 2010) and considered transposable elements (Carrier *et al.* 2012) to determine genetic variability among *V. vinifera* clones, with differences observed between some of the analyzed clones. However, a more efficient and accessible solution for clone identification is yet to be established. Our study used resequencing to identify genetic differences and develop genetic markers for 14 of 18 evaluated clones. The amplicon sequencing technique we implemented enables the parallel evaluation of up to 384 samples, making the diagnostic process more flexible and convenient for the industry. For SB, 16 clonal markers were developed and validated, making differentiating among the 5 evaluated clones possible. In the case of CH, ten clonal markers were validated for 3 out of 4 evaluated clones, allowing differentiation between the 4 clones. The same results were achieved for M, where 3 out of 4 evaluated clones have validated markers that can be combined to differentiate the 4 clones. For CS, 8 clonal markers were validated for 3 out of 5 evaluated clones. The inability to validate markers to identify and individualize some of the clones investigated directly reflects the low genetic variability among grapevine clones.

## Conclusions

The present work significantly improves the grapevine genotyping field by developing a high-throughput amplicon sequencing strategy for clone identification. With the Nextera XT Index Kit v2, this approach enables the simultaneous analysis of up to 384 samples in a single sequencing run, offering a significant advantage over previous SSR-based genotyping methods. The results of this study have the potential to aid in the identification of crucial cultivars and clones used by the global wine industry. Although the study demonstrates the approach's feasibility, it is limited in that the clonal markers were only validated using plant material from the same plants used in the study. The next step should be to validate these markers using grapevine clones from different collections, ideally from nurseries on multiple continents, to show that they can function as clone-specific markers regardless of the source.

This new methodology brings promising advantages to wineries by significantly improving their ability to implement robust tracking protocols for their plant material. By utilizing these markers, wineries can effectively ensure the genetic authenticity of their propagated material, leading to the development of vineyards with greater uniformity and quality.

These markers offer valuable insights from a breeding standpoint by providing clarity on the parentage of selected plants. This information is crucial for breeders, enabling them to make informed decisions regarding the crosses and combinations utilized in their breeding programs. By leveraging these markers, breeders can gain a comprehensive understanding of the genetic relationships between various cultivars, facilitating the development of future grape cultivars with improved and desirable traits.

Overall, utilizing these markers in wineries and breeding programs offers the potential for enhanced quality control, improved tracking of plant material, and greater clarity in parentage determination.

## Supplementary Material

jkad145_Supplementary_Data

## Data Availability

The raw reads for each sample of *Vitis vinifera* clones used during the current study are available in the NCBI repository (BioProject PRJNA847341; https://www.ncbi.nlm.nih.gov/bioproject/PRJNA847341). The raw data for Sauvignon blanc genome assembly used in this work are available in the NCBI repository (BioProject: PRJNA846743; https://www.ncbi.nlm.nih.gov/bioproject/PRJNA846743). VCF files are available in the following: https://doi.org/10.5281/zenodo.7938765. Supplemental material available at G3 online.
