# Supplementary material for: Identification of grapevine clones via high-throughput amplicon sequencing: a proof-of-concept study
Source: G3 (Bethesda). 2023 Jul 3;13(9):jkad145. doi: 10.1093/g3journal/jkad145 (PMC10468313; doi:10.1093/g3journal/jkad145)
Supplement: jkad145_Supplementary_Data [file jkad145_supplementary_data.zip › Supplemental_Material_2_G3-2023-404305.docx]

**Supplementary Material 2. FalconUnzip assembly parameters**

# FalconUnzip parameters ###############################################

length_cutoff = 16526

length_cutoff_pr = 5000

pa_HPCdaligner_option = -k16 -e0.70 -s1000 -t16 -l1000 -h64 -w7 -mtan -mrep2

ovlp_HPCdaligner_option = -k20 -e.96 -s1000 -t32 -l2500 -h256 -mtan -mrep2

pa_DBsplit_option = -x500

ovlp_DBsplit_option = -x500

overlap_filtering_setting = --max_diff 45 --max_cov 45 --min_cov
